# Assessing the Impact of the Otolaryngology Core Curriculum (OCC): A Pre‐ and Post‐Implementation Analysis

**DOI:** 10.1002/ohn.70260

**Published:** 2026-04-30

**Authors:** Madison V. Epperson, Meredith N. Lind, Marc C. Thorne, Robbi A. Kupfer

**Affiliations:** ^1^ Department of Otolaryngology–Head and Neck Surgery University of Michigan Ann Arbor Michigan USA; ^2^ Department of Otolaryngology–Head and Neck Surgery Nationwide Children's Hospital Columbus Ohio USA; ^3^ Present address: Department of Otolaryngology–Head and Neck Surgery Mayo Clinic Rochester Minnesota USA

**Keywords:** curriculum development, flipped classroom, Graduate Medical Education (GME), otolaryngology core curriculum, otolaryngology education, standardized curriculum

## Abstract

**Objective:**

Effective didactic education in otolaryngology residency programs is critical. This study evaluated resident and faculty perceptions before and after (Otolaryngology Core Curriculum) OCC implementation.

**Study Design:**

Prospective cohort study.

**Setting:**

Single Otolaryngology residency program (2023‐2025).

**Methods:**

Anonymous pre‐ and post‐OCC surveys with Likert‐type items were distributed to residents and faculty. Resident surveys assessed weekly didactics preparation time, confidence in otolaryngology knowledge, curriculum effectiveness, comfort identifying educational resources, and perceived exam preparedness. Faculty surveys assessed preparation time, resident knowledge, and engagement. Paired *t*‐tests, Wilcoxon Signed‐Rank, and Mann‐Whitney *U* tests were used for analysis.

**Results:**

24 and 19 residents completed the preimplementation and postimplementation survey, respectively. Weekly resident didactics preparation time increased from 16.3 ± 19.4 minutes to 83.9 ± 49.0 minutes (*P* < .00001). Resident confidence in knowledge improved (*P* = .015) with a large effect size (Cohen's *d* = 0.85; 95% CI [0.07, 1.62]). Perceived didactics effectiveness improved (*P* = .022) with a moderate‐to‐large effect size (Cohen's *d* = 0.79; 95% CI [0.01, 1.55]). Comfort identifying educational resources (*P* = .08) and perceived examination preparedness (*P* = .15) improved, nonsignificantly. Faculty preparation time decreased nonsignificantly, 105.0 ± 33.7 minutes to 90.0 ± 39.7 minutes (*P* = .35). Faculty‐reported resident knowledge improved (*P* = .011) with a large effect size (Cohen's *d* = −1.44; 95% CI [−2.42, −0.43]). Faculty perception of resident engagement improved, nonsignificantly (*P* = .22).

**Conclusion:**

OCC implementation was associated with an increase in resident didactics preparation time, resident perceived knowledge, and faculty perception of resident knowledge, and increased didactics effectiveness.

Effective didactic education is essential for otolaryngology residents to develop foundational knowledge, prepare for board certification, and provide high‐quality patient care. Didactic curricula are increasingly recognized as critical for supporting lifelong learning and specialty‐specific mastery. The Accreditation Council for Graduate Medical Education (ACGME) mandates structured educational experiences across all subspecialties, yet variability in implementation and challenges in delivering structured, engaging didactic content persist across residency programs.

Lecture‐based didactics have long been the mainstay of education, yet their limitations in promoting learner engagement and retention are widely described with increased emphasis on strategies more attuned to modern learners.^1^ The flipped classroom is a key model within this paradigm and has gained significant traction in health professions education, with improvements noted in learner satisfaction, engagement, knowledge acquisition, and retention.^2^, ^3^, ^4^, ^5^ Paralleling the growing evidence to support out‐of‐classroom learning, a plethora of resources have been developed to supplement Otolaryngology education.^6^, ^7^, ^8^ Deciding on and sifting through resources can often overwhelm the time constrained resident. Furthermore, there is a lack of standardization and variability in quality amongst these resources.

In response to these challenges, the Otolaryngology Program Directors Organization (OPDO) and Society of University Otolaryngologists (SUO) endorsed the development of a national Otolaryngology Core Curriculum (OCC),^9^ intended to serve as a shared foundational resource. The OCC aims to promote consistency across programs while offering residents structured review of core topics. Its design encourages flipped‐classroom methodology, where asynchronous, learner‐directed modules are paired with discussion‐based faculty‐facilitated sessions. This model has shown promise in other surgical and medical subspecialties, particularly in promoting engagement and long‐term knowledge retention.^5^, ^8^, ^10^, ^11^, ^12^, ^13^

While the OCC provides structured content, its effectiveness depends on implementation strategy and institutional adaptation. Limited data currently exist on the impact of OCC adoption within residency programs, from the perspectives of both learners and educators. This study aimed to evaluate the effect of OCC implementation within a single academic otolaryngology residency program. Specifically, we sought to assess changes in resident engagement, preparation habits, perceived curriculum effectiveness, and faculty perceptions of resident knowledge and engagement. By capturing both quantitative and qualitative feedback before and after curriculum adoption, we aimed to understand how OCC integration impacts the learning environment and identify areas for optimization.

## Methods

### Study Design

A prospective cohort study was conducted at a single tertiary academic otolaryngology training program over 2 academic years (2023‐2025) to evaluate the impact of the novel OCC on resident and faculty perceptions of didactic education. The University of Michigan Institutional Review Board deemed this study exempt (HUM00066405).

### Participants

Participants included otolaryngology residents (PGY‐1 through PGY‐5) and full‐time faculty affiliated with the residency program. All residents and faculty were invited to participate via email. Residents or faculty who did not participate in both preintervention and postintervention survey phases were excluded from paired analyses but included in unpaired comparisons where appropriate.

### Intervention

Prior to the OCC, this institution, utilized a traditional faculty‐led weekly lecture system. Cases were occasionally incorporated, but this was faculty‐dependent. Likewise, some faculty incorporated questions throughout their lectures, but this was highly variable. The OCC was implemented in July 2024 and featured a structured, flipped‐classroom format. Weekly modules were assigned with curated pre‐reading per the module and a quiz. The didactics chief resident selected modules and appropriate faculty moderators such that there would be 1 module per week, rarely 2 if topics coincided. Faculty were then able to pick the date that worked best for their schedule. Modules were selected from the 25 modules released the first 6 months of the OCC. The OCC will eventually consist of 100 modules in total, released over a 2‐year period. The didactic sessions were led by faculty and the PGY‐5 didactics chief resident. They followed a case‐based, discussion‐oriented format, with cases provided as part of the OCC. Program expectations for the faculty and residents are depicted in Table 1. Time was allotted for review of questions related to the quiz. The intervention spanned 1 academic year and replaced the prior traditional weekly lecture by faculty.

**Table 1:** Faculty and Resident Expectations

	Faculty expectations	Resident expectations
Before the session	Review the OCC “Overview” and “Conference Prep” materials	Read the module. Look over “Conference Prep” cases if you choose
	Identify key learning points to emphasize during the discussion	Take the self‐assessment quiz
During the session	*In‐person* moderator. Facilitate discussion. Encourage broad resident participation.	Actively participate. Take advantage of this as learning time, minimizing clinical work

### Survey Instruments

Anonymous surveys were developed for both residents and faculty using an iterative consensus process. All subjective items were measured using a 5‐point Likert‐type scale. Surveys also included optional free‐text responses for qualitative analysis.

Resident survey domains included:

- Weekly didactic preparation time (minutes).
- Comfort identifying educational resources.
- Perceived exam preparedness.
- Confidence in otolaryngology knowledge.
- Perceived effectiveness of the didactic curriculum.

Faculty surveys assessed:

- Weekly faculty preparation time (minutes).
- Perception of resident knowledge.
- Perception of resident engagement during the session.

### Data Collection

Surveys were distributed electronically using REDCap (Research Electronic Data Capture) prior to OCC implementation (Spring 2024, in reference to 2023‐2024 academic year) and at the end of the first‐year postimplementation (Spring 2025, in reference to 2024‐2025 academic year). Responses were matched anonymously for paired analyses using unique identifiers generated for participants.

### Statistical Analysis

Descriptive statistics were calculated for all variables. Paired *t*‐tests were used to compare resident weekly preparation time. Independent *t*‐tests were used to compare faculty preparation time. Wilcoxon signed‐rank tests were used for paired analyses of resident ordinal data including confidence in otolaryngology knowledge, perceived effectiveness of the didactic curriculum, comfort identifying educational resources, and perceived preparedness for in‐training or board examinations. Mann‐Whitney *U* tests were used to compare unpaired faculty‐reported outcomes including resident knowledge and resident engagement. Effect sizes were calculated using Cohen's *d* with 95% confidence intervals (CI) reported. A *P* < .05 was considered statistically significant. Analyses were performed using Stata 18 (StataCorp LLC). Qualitative data were summarized.

## Results

A total of 24 residents completed the pre‐OCC survey and 19 completed the post‐OCC survey. 14 residents completed both surveys and were included in paired analyses. 11 faculty completed the presurvey and 9 completed the postsurvey.

### Resident Outcomes

**Figure 1:**
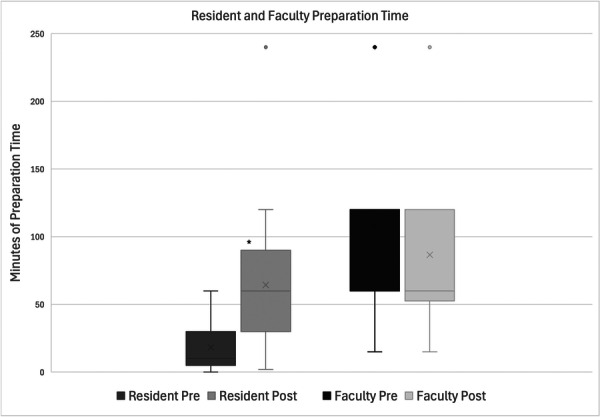
This plot depicts minutes per week of preparation time for residents and faculty, demonstrating the significantly increased resident preparation time post implementation of the Otolaryngology Core Curriculum and unchanged faculty preparation time. (* indicates significance).

**Figure 2:**
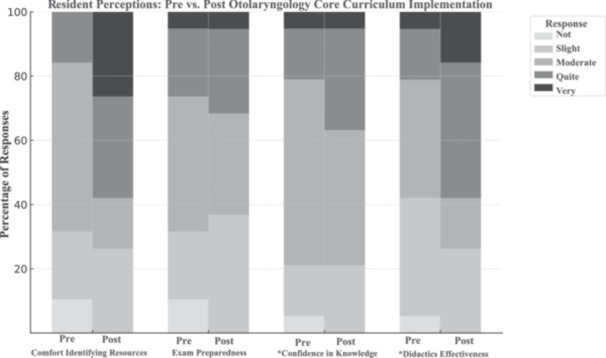
This plot depicts resident perceptions pre‐ and post‐Otolaryngology Core Curriculum Implementation. Confidence in Otolaryngology knowledge and didactics effectiveness were significantly improved post implementation. (* indicates significance).

Resident‐reported weekly preparation time for didactics significantly increased (Figure 1) following OCC implementation, from a mean of 16.3 ± 19.4 minutes to 83.9 ± 49.0 minutes (*P* < .00001), reflecting a substantial increase in self‐directed engagement. Resident confidence in otolaryngology knowledge significantly improved (*P* = .015) with a large effect size (Cohen's *d* = 0.85; 95% CI [0.07, 1.62]). Similarly, perceived effectiveness of the didactic curriculum improved significantly (*P *= .022) with a moderate‐to‐large effect size (Cohen's *d* = 0.79; 95% CI [0.01, 1.55]). While improvements were observed in resident comfort identifying educational resources (*P* = .081) and perceived preparedness for in‐training or board examinations (*P *= .152), these changes were not statistically significant. Results pictured in Figure 2.

### Faculty Outcomes

**Figure 3:**
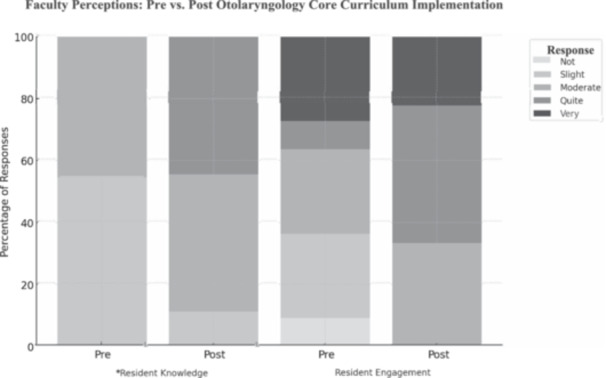
This plot depicts faculty perceptions of residents pre‐ and post‐Otolaryngology Core Curriculum Implementation. Faculty perception of resident knowledge significantly improved post implementation. (* indicates significance).

Faculty‐reported preparation time for teaching decreased (Figure 1) from 105.0 ± 33.7 minutes to 90.0 ± 39.7 minutes, though this difference did not reach statistical significance (*P *= .35). Faculty perception of resident knowledge significantly improved post‐OCC (*P* = .011). The effect size was large (Cohen's *d* = −1.44; 95% CI [−2.42, −0.43]), indicating a meaningful improvement in faculty ratings. Faculty perception of resident engagement improved from a median of “moderately engaged” to “quite engaged,” but the change was not statistically significant (*P* = .22). Results pictured in Figure 3.

### Qualitative Feedback

Free‐text responses from residents and faculty highlighted multiple benefits of the OCC. A few representative quotes are listed in Appendix 1. Common themes included increased structure and predictability, improved session quality due to flipped‐classroom preparation, and enhanced engagement during discussions. Residents appreciated having program‐provided resources with an outlined schedule. Faculty noted that the assigned materials reduced the burden of content preparation and better focused resident attention. Several residents cited appreciation for having clearly defined learning objectives. Residents also positively reviewed the links to additional resources and content, such as surgical videos or past AAO‐HNS Annual Meeting lectures. Feedback indicated room for improvement on quiz question quality to better align with module content. Graphic clarity was also flagged for improvement.

## Discussion

In response to the growing call for a standardized curriculum incorporating evidence‐based modernized learning strategies, the OCC was developed.^9^ It aims to promote consistency across programs, designed with intentionality to encourage flipped‐classroom methodology and to permit asynchronous learning, known to be increasingly desired and utilized by otolaryngology residents.^6^, ^8^ In this study, the first to evaluate the national OCC, we sought to assess the impact of OCC adoption on faculty and residents alike. We found that OCC implementation was associated with a significant increase in resident didactics preparation time and a substantial improvement in resident perceived otolaryngology knowledge. Residents found the new curriculum to be more effective than the traditional curriculum. Faculty perception of resident knowledge improved.

This model has shown promise in other surgical and medical subspecialties, particularly in promoting engagement and long‐term knowledge retention.^5^, ^11^, ^12^, ^14^, ^15^, ^16^, ^17^, ^18^ In general surgery, the development and implementation of a standardized curriculum, Surgical Counsel on Resident Education (SCORE), has improved in‐training‐exam (ITE) performance, resident accountability, and well‐being.^11^, ^19^, ^20^ Of note, the purpose of the OCC is to support programs and faculty by providing a learning platform that promotes a strong foundation of core knowledge required for otolaryngology practice. As such, module topics were selected by a group of otolaryngology residency program directors, and modules were created by groups of content experts with a rigorous peer review process. By definition, module topics will align with the American Board of Otolaryngology‐Head and Neck Surgery Otolaryngology Training Exam/ Written Qualifying Exam Blueprint, but they are specifically not meant to serve as “board preparation” materials. While analysis is not yet available, evaluation of objective trainee outcomes such as ITE scores and board examination pass rates will be important in the future as an objective OCC quality metric.

The advantages of e‐learning in the setting of surgery are well established. In addition to being highly accessible and updatable, e‐learning platforms can accommodate a broad variety of learning styles and are effective in teaching an array of surgically relevant information. Interactive, Web‐based media have been shown not only to significantly improve surgical skill, but also to reduce error rates and operative time in both general surgery and plastic surgery residents.^16^, ^17^ The use of case‐based e‐learning software compared to traditional didactics models has been documented to significantly improve knowledge retention in medical students.^13^

Within otolaryngology, there is a small foundation of literature to support use and implementation of asynchronous learning resources and the flipped classroom model. A systematic review found e‐learning to be strongly desired by otolaryngology residents with increased knowledge compared to traditional classroom models.^8^ In 2020, Kohler et. al surveyed otolaryngology program directors and found that while program directors value the flipped classroom principles, only 37.8% of programs used the flipped classroom. They found that barriers such as faculty time, resident accountability, and resource standardization limited widespread implementation.^21^ Moreover, Malka et al found that the use of asynchronous resources for resident education is increasing, currently outpacing that of textbook and traditional didactics education. They use these findings to comment on the need for development of high‐quality multimedia resources.^6^ Our findings suggest that OCC's structured flipped classroom model—with weekly multimedia content curated from experts in the field—may address this gap and provide an accessible framework for broader adoption.

Our study is in agreement with prior studies suggesting positive resident perceptions of nontraditional, flipped classroom, web‐based learning platforms. Salmon et al demonstrated that a novel flipped classroom curriculum designed and implemented at a single institution was viewed favorably by the residents with increased knowledge retention.^22^ Similarly, our study found favorable review by the residents, with improved confidence in otolaryngology knowledge and perceived increased effectiveness of the curriculum compared to the traditional model. Importantly, the structure of flipped learning promotes accountability and preparation, which may explain the significant increase in resident preparation time observed in our study. This is concordant with the SCORE platform, noting increased resident accountability.^19^ One potential barrier to the flipped classroom model is faculty acceptance with concern for lengthened faculty preparation time.^21^ Our study demonstrates that the concern of faculty preparation time may be ameliorated by a structured curriculum, as we found that faculty preparation time was less, not significantly so, than with a traditional didactics format.

Qualitative comments in our study reinforced the quantitative findings. Residents cited the flipped format, consistent structure, and collaborative nature of sessions as key strengths. Faculty appreciated the ability to focus on discussion and higher‐order questions rather than content creation. These sentiments echo findings from McLaughlin et al and King et al, both of which highlighted increased learner engagement and satisfaction with flipped classroom formats in graduate medical education.^3^, ^10^

Our results should be interpreted considering several limitations. This was a single‐institution study which may limit generalizability as OCC implementation may vary based on program size, available faculty resources, or resident didactic culture. For instance, at this institution, every session was co‐led by faculty and the didactics chief resident with 1.5 hours of weekly protected time for the session. This is not likely the identical model for all programs. Nevertheless, a benefit of the OCC compared to traditional lecture‐based models is the standardized, expert‐curated, identical content provided to all programs, despite the *in‐person* session variability. Additionally, incomplete paired responses introduce potential response bias. Lastly, the use of subjective, self‐reported survey data, while valuable in capturing perspectives, may not fully reflect objective improvements in knowledge or performance.

Despite these limitations, our study has several notable strengths. It is the first formal evaluation of the OCC and leverages a prospective, preintervention and postintervention design with input from both residents and faculty. The inclusion of effect size analysis underscores the practical importance of changes observed in resident confidence, preparation time, and perceived effectiveness. Still, they should be interpreted with caution given the small sample size and variability in survey responses. Future directions include multi‐program studies to assess reproducibility and generalizability. Incorporating objective outcomes like ITE scores may strengthen conclusions. Lastly, applying a structured curriculum evaluation framework, such as Kirkpatrick's model, would further guide OCC refinement and long‐term impact assessment.

In summary, our study supports the growing body of evidence that flipped classroom and structured curricula, when thoughtfully implemented, can improve faculty and learner satisfaction with curricula and outcomes. As otolaryngology continues to evolve educationally, OCC represents a promising step toward national curricular standardization and pedagogical modernization.

## Author Contributions

**Madison Epperson**, design, conduct, analysis, presentation of the research; **Meredith N. Lind**, design, conduct; **Marc C. Thorne**, design, analysis; **Robbi A. Kupfer**, design, conduct.

## Disclosures

### Competing interests

None.

### Funding source

None.

## Supporting information

Appendix 1. Representative Quotes from Residents and Faculty. Appendix 1 demonstrates representative quotes from residents and faculty to provide qualitative insight into the Otolaryngology Core Curriculum.
