# Supplementary material for: Assessing the Impact of the Otolaryngology Core Curriculum (OCC): A Pre‐ and Post‐Implementation Analysis
Source: Otolaryngol Head Neck Surg. 2026 Apr 30;175(2):357–63. doi: 10.1002/ohn.70260 (PMC13418059; doi:10.1002/ohn.70260)
Supplement: Supplementary file 1 — Appendix 1. Representative Quotes from Residents and Faculty. Appendix 1 demonstrates representative quotes from residents and faculty to provide qualitative insight into the Otolaryngology Core Curriculum. [file OHN-175-357-s001.docx]

Appendix 1.

| Resident- “I appreciate having set content to get through every week that is presented in a succinct but thorough way with links to videos and content to dive deeper if I have time.” |
| --- |
| Resident- “With the OCC, I don’t have to waste time sifting through a bunch of different resources. This way I can spend more time on the actual content.” |
| Resident- “I like the in-person discussion where I can bring up my questions on the topic to senior residents and faculty” |
| Faculty- “Really loved the interactive and case-based nature of this session. The written content was concise yet comprehensive, and it was really nice to have questions to review with the residents during the session. I like that I did not have to lecture to the residents for 1-1.5 hours.” |
| Faculty- “I liked the fact that all of the material was easily available. I liked that the residents took responsibility for helping to organize the discussion. I felt there was increased engagement.” |
